# Evaluation of clinical relevance of weight nadirs and weight regain during 3 years of treatment with tirzepatide in participants with obesity or overweight and prediabetes

**DOI:** 10.1016/j.obpill.2026.100333

**Published:** 2026-09-18

**Authors:** Louis J. Aronne, Royce P. Vincent, Klara R. Klein, Julia Fraseur Brumm, Irina Jouravskaya, Dachuang Cao, Georgios K. Dimitriadis, Julia P. Dunn

**Affiliations:** a Comprehensive Weight Control Center, Weill Cornell Medicine, New York, USA; b King's College Hospital NHS Foundation Trust, London, UK; c University of North Carolina at Chapel Hill, North Carolina, USA; d Eli Lilly and Company, Indianapolis, USA

**Keywords:** Prediabetes, SURMOUNT-1 3-year, Tirzepatide, Weight nadir, Weight regain

## Abstract

**Objective:**

For adults with obesity, fluctuations in body weight are expected to occur during weight management. Weight nadirs, which can become a point of focus for people living with obesity, are often hard to maintain. This study aimed to investigate the not well-defined clinical relevance of weight nadir, especially during long-term treatment with an obesity medication.

**Methods:**

This was a post hoc analysis of the phase 3, double-blind, randomized, placebo-controlled SURMOUNT-1 3-year clinical trial in participants with obesity or overweight and at least one weight-related complication. The analysis included participants who were tirzepatide-adherent (≥75% of doses received) with obesity, or overweight, and prediabetes at randomization and had ≥5% weight reduction from baseline at time of weight nadir. Weight regain analyses from weight nadir to Week 176 included mean percent change, and participants were grouped as having <5%, 5% to <10%, and ≥10% weight regain.

**Results:**

In this post hoc analysis, the mean weight regain from nadir weight to Week 176 was 3.8 kg, 4.4 kg, and 3.7 kg for those treated with tirzepatide 5 mg, 10 mg, and 15 mg, respectively. For the pooled tirzepatide doses, 70% of participants had <5% weight regain, 22% of participants had 5 to <10% weight regain, and 8% of participants had ≥10% weight regain. The median percent of weight reduction maintenance from time of weight nadir to Week 176 was 94.8%, 69.9%, and 44.3%, for <5%, 5-<10%, and ≥10%, respectively. While at baseline 94% of tirzepatide-treated participants had high waist-to-height ratio (WHtR) (>0.59), at week 176 this had reduced to 45%, 59%, and 68% in the <5%, 5% to <10%, and ≥10% weight regain groups, respectively. At Week 176, conversion from prediabetes to normoglycemia occurred in over 90% of tirzepatide-treated participants regardless of weight regain.

**Conclusion:**

These findings suggest that most tirzepatide-treated participants with baseline prediabetes in the SURMOUNT-1 3-year study had limited weight regain after reaching weight nadir and sustained improvements in glycemia (SURMOUNT-1 ClinicalTrials.gov identifier: NCT04184622.).

**Clinical relevance:**

In the current study, 92% of prediabetes participants treated with tirzepatide in the SURMOUNT-1 3-year study had a stable weight journey. Their limited weight regain from weight nadir appears not to be clinically meaningful, as improvements in cardiometabolic risk parameters were largely maintained.

## Graphical abstract

**Figure:**
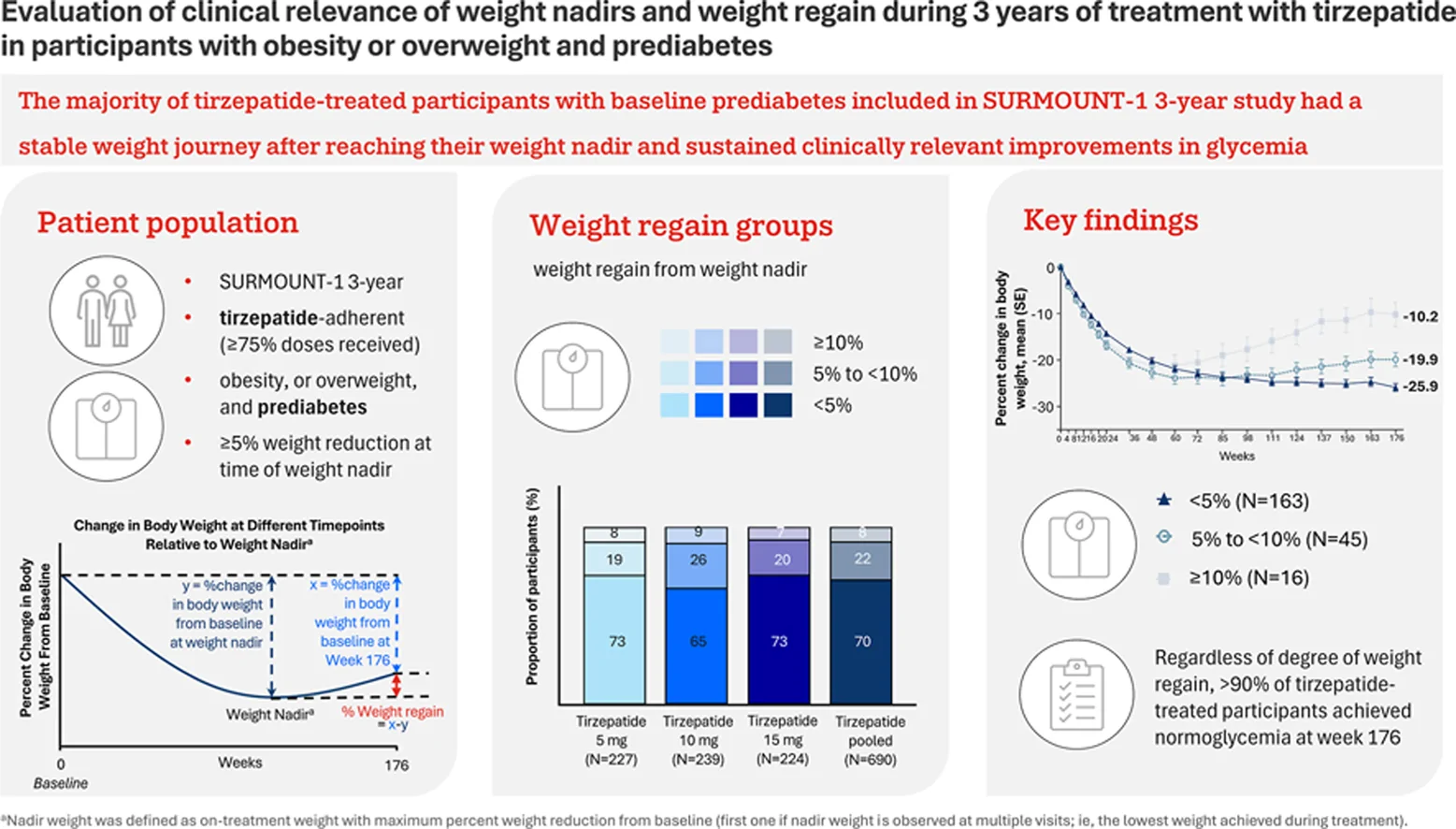

## 1 Introduction

Obesity is a chronic, progressive disease that requires long-term medical management. Substantial weight regain with loss of cardiometabolic benefit has been demonstrated with discontinuation of obesity medications (OM), including tirzepatide [[1], [2], [3], [4]]. However, less is understood regarding changes in weight while on treatment with an OM. In chronic disease management with pharmacologic therapeutics of many diseases, such as hypertension [5], diabetes [6], and rheumatoid arthritis [7], treatment effect often attenuates with time. For some therapies, loss of response develops despite continued treatment (secondary failure) [8,9]. Understanding treatment effect over time of OM would inform clinical management of overweight and obesity.

When treating obesity, all people will experience a weight nadir at one time or another. It is not uncommon that weight nadir can become a distraction to people during weight maintenance as they become focused on numerical weight changes as opposed to long-term health improvements [10]. However, the clinical relevance of the level of weight regain from weight nadir is not established, limiting clinical guidance. The current post hoc analysis of the SURMOUNT 1 3-year study evaluated weight nadirs and the association of degree of weight regain from weight nadir and cardiometabolic risk parameters in participants treated with tirzepatide, a dual glucose-dependent insulinotropic polypeptide and ​glucagon-like peptide-1 receptor agonist. Those participants treated with tirzepatide experienced a mean percent change in body weight of −12.3% to −19.7% across tirzepatide doses compared to −1.3% with placebo at the end of SURMOUNT-1 3-year study. Over 90% of tirzepatide participants achieved and maintained normoglycemia at end of the study compared to 59% of placebo and 1.3% compared with 13.3% developed type 2 diabetes, respectively [11].​ The current study aims to further support clinical decision-making by providing insights beyond mean effects.

## 2 Methods

### 2.1 Study design and subjects

This was a post hoc analysis of the phase 3, double-blind, randomized, placebo-controlled SURMOUNT-1 3-year clinical trial in participants with obesity or overweight with at least one weight-related complication. All participants at baseline had prediabetes as defined by the 2019 American Diabetes Association Standards of Medical Care in Diabetes (N = 1032; n = 270 placebo, n = 762 tirzepatide) [11]. The definition indicates the following parameters for prediabetes: Fasting glucose is 100–125 mg/dL (5.6–6.9 mmol/L), 2H glucose is 140-199 mg/dL (7.8–11.0 mmol/L), and Hemoglobin A1c (HbA1c) is 5.7%–6.4% (39–47 mmol/mol). The key inclusion criteria were adults with obesity (body mass index [BMI] ≥30 kg/m^2^), or overweight (BMI ≥27 kg/m^2^) with ≥1 weight-related comorbidities (hypertension, dyslipidemia, obstructive sleep apnea or cardiovascular disease). Prediabetes at randomization was an eligibility requirement for the 3-year portion of the study. Key exclusion criteria were type 1 or type 2 diabetes, >5 kg body weight change within ​90 days before screening, prior or planned surgical treatment for obesity​, and treatment that promotes weight loss within 90 days before screening. Participants were randomly assigned in a 1:1:1:1 ratio to once weekly 5, 10, or 15 mg tirzepatide or matched placebo. Participants with prediabetes were followed for 176 weeks, followed by a 17-week off-treatment period.

This post hoc analysis of the SURMOUNT-1 3-year study included 690 tirzepatide-adherent participants (≥75% of doses received) who had ≥5% weight reduction at time of weight nadir. This represents 90.6% of all the tirzepatide-treated participants with prediabetes (n = 762).

### 2.2 Details to identifying individual weight nadir, waist-to-height ratio (WHtR), and cardiometabolic risk parameters

Individual participant weight nadir was defined as on-treatment weight with maximum percent weight loss from baseline (first one if weight nadir was observed at multiple visits) (ie, the lowest weight achieved during treatment) (Supplementary Fig. 1). Weight regain from weight nadir to Week 176 was defined as the difference between percent weight reduction from baseline to nadir and percent weight reduction from baseline to Week 176. Thus, weight regain from weight nadir was referring to a percent of pretreatment body weight.

WHtR risk category was used as an assessment of central adiposity, an established cardiometabolic risk parameter [12]. Since waist circumference (WC) was measured at all time points that body weight was measured, WHtR category was the primary cardiometabolic risk parameter of interest since timing could be directly paired with weight nadir.

We also explored in a descriptive manner other cardiometabolic risk parameters (systolic blood pressure, diastolic blood pressure, HbA1c, fasting serum glucose, and lipids) for how weight regain from weight nadir was associated with change in earlier benefits to end of study. As these measures were not collected at all time points that body weight was measured, the effect was evaluated by comparing baseline, Week 72 and Week 176 assessments.

### 2.3 Statistical analysis

Analyses used the on-treatment data of the 690 tirzepatide-adherent participants (≥75% of doses received) who had ≥5% weight reduction at time of weight nadir. Time to weight nadir, percent weight change at weight nadir, percent weight change at Weeks 176, and weight regain were summarized descriptively using mean and standard deviation (SD) by tirzepatide dose. Last observation carried forward (LOCF) was applied if Week 176 observation was missing.

Participants were also categorized as <5%, 5% to <10% and ≥10% weight regain groups, either with all tirzepatide doses pooled or by separate dose. Mean weight regain and proportion of participants who had WHtR categorical shift from weight nadir to Week 176 were summarized descriptively. WHtR risk categories were defined as healthy (WHtR ≤0.49), increased (WHtR >0.49 to ≤0.59), or high (WHtR >0.59)​ [12]. Time course of other cardiometabolic parameters (e.g., blood pressure and lipids) were summarized using mixed model for repeated measures (MMRM), and model-based estimate (MBE) and standard error (SE) were reported.

## 3 Results

### 3.1 Baseline characteristics

The mean (SD) age of the participants was 48.5 years (11.7). Most participants were women (64.6%) and White (74.2%). Participants had a mean (SD) body weight of 107.0 kg (23.5), BMI of 38.6 (7.0), and waist circumference of 116.1 cm (15.3) at baseline (Table 1, Supplementary Table 1). The average duration of obesity was reported as 15.5 (11.1) years, and 42.2% of the participants had at least two obesity-related complications (Supplementary Table 2).

**Table 1:** Demographic and clinical characteristics of the participants at baseline.

Characteristic	% Weight Regain from Baseline <5% (N = 484)	% Weight Regain from Baseline 5-<10% (N = 149)	% Weight Regain from Baseline ≥10% (N = 57)	Total (n = 690)
Age, years	48.8 (11.8)	49.2 (10.6)	44.3 (12.9)	48.5 (11.7)
Female sex, n (%)	310 (64.0)	94 (63.1)	42 (73.7)	446 (64.6)
Race or ethnic group, n (%)^a^				
American Indian or Alaska Native	36 (7.4)	12 (8.1)	6 (10.5)	54 (7.8)
Asian	59 (12.2)	5 (3.4)	4 (7.0)	68 (9.9)
Black or African American	33 (6.8)	10 (6.7)	2 (3.5)	45 (6.5)
Multiple	4 (0.8)	1 (0.7)	3 (5.3)	8 (1.2)
Native Hawaiian or other Pacific Islander	3 (0.6)	0 (0)	0 (0)	3 (0.4)
White	349 (72.1)	121 (81.2)	42 (73.7)	512 (74.2)
Weight, kg	107.0 (23.5)	106.6 (24.0)	108.3 (21.8)	107.0 (23.4)
body-mass index, kg/m^2^	38.6 (7.1)	38.3 (6.9)	39.8 (6.4)	38.6 (7.0)
Waist Circumference, cm	115.9 (15.2)	116.4 (16.2)	117.7 (14.5)	116.1 (15.3)
Waist-to-height ratio	0.7 (0.1)	0.7 (0.1)	0.7 (0.1)	0.7 (0.1)
Waist-to-height ratio category, n (%)^&^				
>0.49 - ≤0.59	34 (7.0)	11 (7.4)	1 (1.8)	46 (6.7)
>0.59	450 (93.0)	137 (92.6)	56 (98.2)	643 (93.3)
Treatment, n (%)				
TZP 5 mg	165 (34.1)	43 (28.9)	19 (33.3)	227 (32.9)
TZP 10 mg	156 (32.2)	61 (40.9)	22 (38.6)	239 (34.6)
TZP 15 mg	163 (33.7)	45 (30.2)	16 (28.1)	224 (32.5)
Blood pressure, mmHg				
Systolic	126.2 (12.6)	126.5 (13.6)	124.5 (10.9)	126.1 (12.7)
Diastolic	80.4 (8.3)	80.9 (8.2)	80.7 (7.5)	80.6 (8.2)
HbA1c (%)	5.8 (0.4)	5.8 (0.3)	5.8 (0.3)	5.8 (0.3)
Fasting Serum Glucose, mg/dL	101.5 (9.2)	101.6 (10.2)	100.5 (10.1)	101.5 (9.5)

Data are mean (SD) unless otherwise noted. HbA1c = Hemoglobin A1c; SD = standard deviation; TZP = tirzepatide.

& Waist-to-height ratio category 0–0.5 had 0 participants in all weight regain groups.

a Race or ethnic group was reported by the participants.

### 3.2 Weight reduction at nadir and weight regain from nadir at week 176

Across the 3 doses of tirzepatide, 5 mg, 10 mg, and 15 mg, the mean percent weight change (SD) at time of weight nadir was −19.0 (8.4)%, −24.3 (10.7)%, and −26.0 (10.1)%, respectively, and at Week 176, −15.5% (9.0), −20.1% (11.3) and −22.5% (10.8), respectively (Fig. 1). The mean weight regain from nadir weight to Week 176 was 3.8 kg, 4.4 kg, and 3.7 kg for those treated with tirzepatide 5 mg, 10 mg, and 15 mg, respectively. The change from time of weight nadir to Week 176 was due to a mean (SD) weight regain of 3.6% (3.9), 4.1% (4.8), and 3.5% (4.1) from baseline, respectively, for tirzepatide 5 mg, 10 mg, and 15 mg, respectively (Fig. 1). The randomized doses of tirzepatide were received as the highest dose by 243 (98.4%), 246 (93.9%), and 229 (90.5%) participants during the 176 weeks, respectively for the 5 mg, 10 mg, and 15 mg tirzepatide doses, and 237 (96.0%), 224 (85.5%), and 197 (77.9%) participants received the randomized as the final dose for the 5 mg, 10 mg, and 15 mg tirzepatide dose respectively. The proportion of participants in the 3 groups for weight regain from weight nadir was similar for each tirzepatide dose. The mean (SD) time to weight nadir was 21.1 (11.4) months, 21.1 (11.6) months, and 22.6 (11.5) months for those treated with tirzepatide 5 mg, 10 mg, and 15 mg, respectively. More than a third (48.9%, 46.4% and 38.8% of the analysis population receiving 5 mg, 10 mg, and 15 mg tirzepatide, respectively) of participants had reached their weight nadir by Week 72 (inclusive). More than half (51.1%, 53.6% and 61.2% of the analysis population receiving 5 mg, 10 mg and 15 mg tirzepatide, respectively) of participants reached their weight nadir after Week 72.

**Fig. 1:**
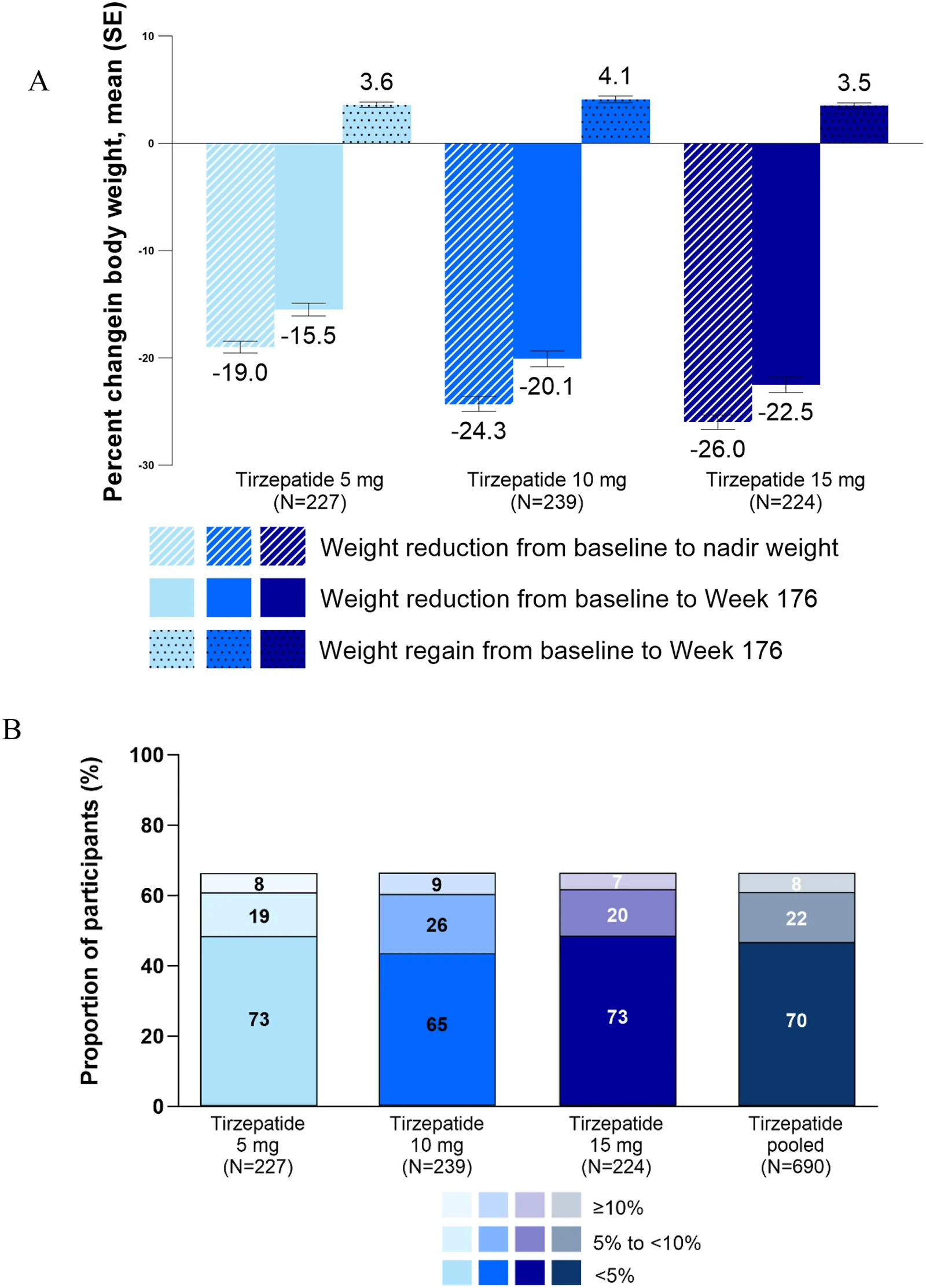
A. Percent change in body weight by tirzepatide dose. **B.** Proportion of participants experiencing various levels of weight regain from weight nadir to Week 176 by tirzepatide doses. Percent weight reduction from baseline to Week 176 was determined using LOCF. LOCF = last observation carried forward.

For the pooled tirzepatide doses, 70% of participants had <5% weight regain, 22% of participants had 5 to <10% weight regain, and 8% of participants had ≥10% weight regain. (Fig. 1, BMI see Supplementary Table 3). In Fig. 2, the percent weight change curves for tirzepatide 15 mg demonstrate that participants had MBE (SE) body weight reductions of 25.9% (0.84), 19.9% (1.52), and 10.2% (2.62) from baseline at Week 176 in the <5%, 5% to <10%, and ≥10% weight regain groups, respectively. The curves for 5 mg and 10 mg by weight regain group are included in the supplementary appendix (Supplementary Fig. 2). In participants receiving tirzepatide at Week 176, mean body weight pooled across doses was MBE (SE) 83.1 kg (0.59), 90.1 kg (1.01), and 96.3 kg (1.65) for the <5%, 5% to <10%, and ≥10% weight regain groups, respectively (Supplementary Fig. 3, Supplementary Table 4).

**Fig. 2:**
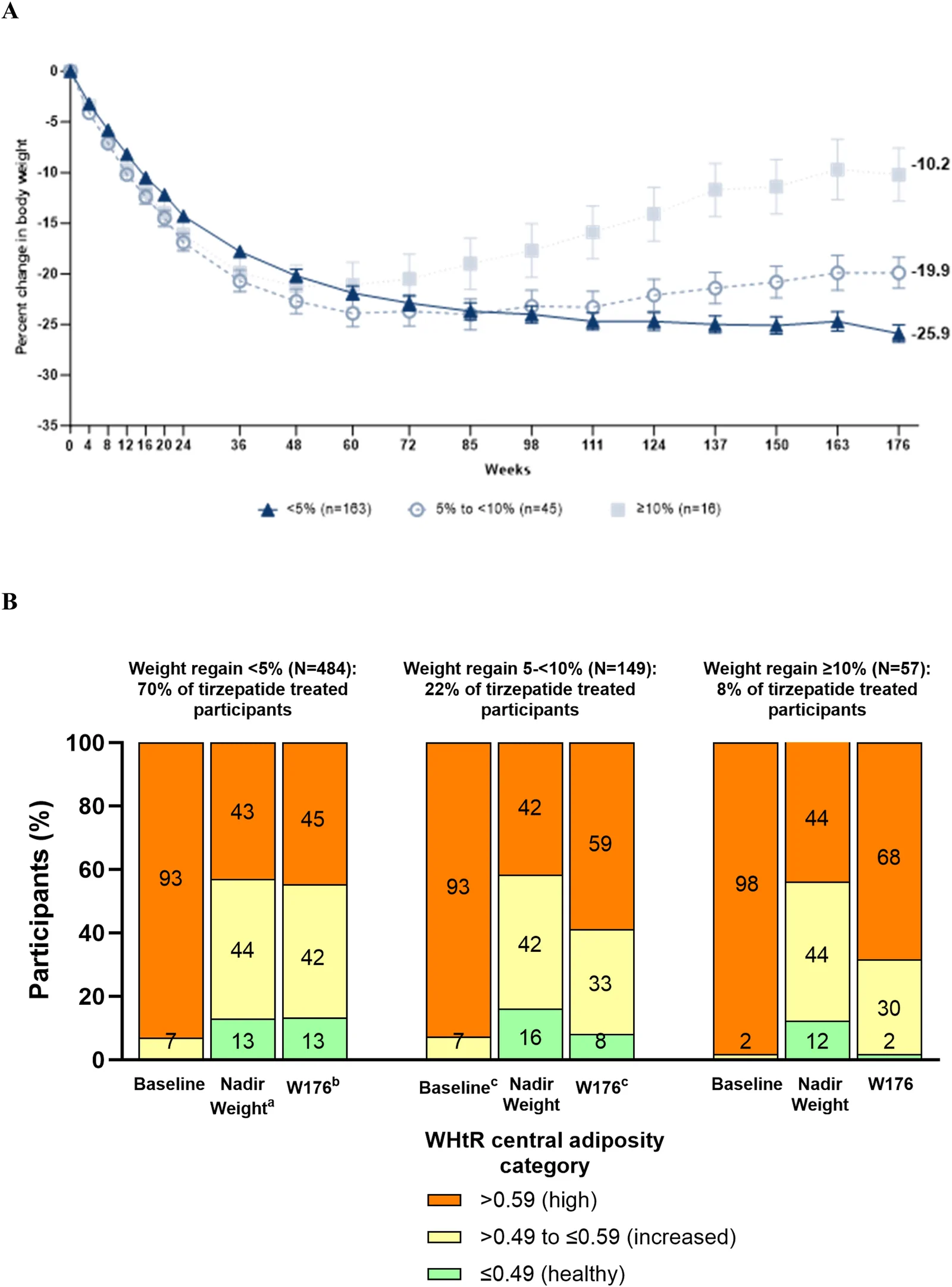
A. Percent change in body weight by weight regain group from baseline to Week 176 in participants receiving tirzepatide 15 mg. Data are MBE (SE). Statistical analyses were performed using MMRM with model terms of baseline, country, sex, time, treatment, and treatment-by-time interaction. **B.** WHtR category at baseline, weight nadir, and Week 176 by level of weight regain for pooled tirzepatide.^a^N = 483; ^b^N = 482; ^c^N = 148. Data shown are based on tirzepatide-adherent participants (received ≥75% of doses) with prediabetes at randomization during Weeks 0–176 and ≥5% weight loss at weight nadir.​ MMRM = mixed model for repeated measures; MBE = model-based estimate; SE = standard error; TZP = tirzepatide; WHtR = waist-to-height-ratio; W = Week.

For the three weight regain groups, the median percent of weight reduction maintained from time of weight nadir to Week 176, was 94.8%, 69.9% and 44.3%, for <5%, 5-<10%, and ≥10%, respectively. The median (interquartile range) weight regain was 1.2 (0.0–2.9), 7.2 (5.8–8.9), and 13.7 (11.8–16.6) kg, for <5%, 5-<10%, and ≥10%, respectively (Table 2). Accordingly, as the amount of weight regain increased the proportion with worsening in WHtR category from time of weight nadir to Week 176 increased, 6.4%, 24.3% and 36.8%, for the three weight regain groups, respectively.

**Table 2:** Week 176 Clinical characteristics of weight regain from weight nadir group.

Regain from weight nadir; % calculated from baseline weight	Sample size	Median (IQR) of weight regain kg from nadir to Week 176	Median (IQR) Percent of weight change from baseline to time of weight nadir that is maintained at Week 176	Median (IQR) of change in waist circumference cm, BL to Week 176	Median (IQR) of change WHtR ratio (as a continuous measure) From BL to Week 176	Proportion with worsening in WHtR ratio category from Weight nadir to Week 176, n (%)	Proportion with normoglycemia at week 176, n (%)
<5%	n = 484 (70%)	1.2 (0.0–2.9)	94.8 (86.8–100.0)	−17.0 (−25.3–−10.0)	−0.10 (−0.15–−0.06)	31 (6.4)	465 (96.1)
5-<10%	n = 149 (22%)	7.2 (5.8–8.9)	69.9 (54.6–79.0)	−13.8 (−21.5–−8.0)	−0.09 (−0.13–−0.05)	36 (24.3)	138 (92.6)
≥10%	n = 57 (8%)	13.7 (11.8–16.6)	44.3 (28.1–56.3)	−11.0 (−19.0–−4.8)	−0.07 (−0.12–−0.03)	21 (36.8)	52 (91.2)
All	n = 690 (100%)	2.7 (0.2–6.0)	88.5 (72.5–99.0)	−15.5 (−23.5–−9.3)	−0.10 (−0.15–−0.06)	88 (12.8)	655 (94.9)

Data are median (interquartile range) unless otherwise noted. BL = baseline; IQR = interquartile range; WHtR = waist-to-height ratio.

### 3.3 Weight regain from weight nadir and WHtR central adiposity category

At baseline, approximately 94% of participants had high WHtR (>0.59), including 93%, 93%, and 98% in the <5%, 5% to <10%, and ≥10% weight regain from weight nadir groups, respectively (Fig. 2). At time of weight nadir, the proportion with high WHtR had reduced to 43%, 42%, and 44%, while by the end of the study 45%, 59%, and 68% of participants had high WHtR in the <5%, 5% to <10%, and ≥10% weight regain from nadir groups, respectively.

### 3.4 Other cardiometabolic measures in weight regain from nadir groups

Mean systolic blood pressure (SBP) and diastolic blood pressure (DBP) were lower at Week 176 compared to baseline in all groups across doses except for those treated with tirzepatide 10 mg, who had weight regain ≥10% (Fig. 3 and Supplementary Table 5). The SBP and DBP reductions seen at Week 72 were mostly sustained at week 176 in those with <5% and 5% to <10% weight regain. At Week 176, in those who regained >10%, there were attenuations of the improvements in SBP and DBP that occurred at Week 72 (Supplementary Tables 5, 6, and 7).

**Fig. 3:**
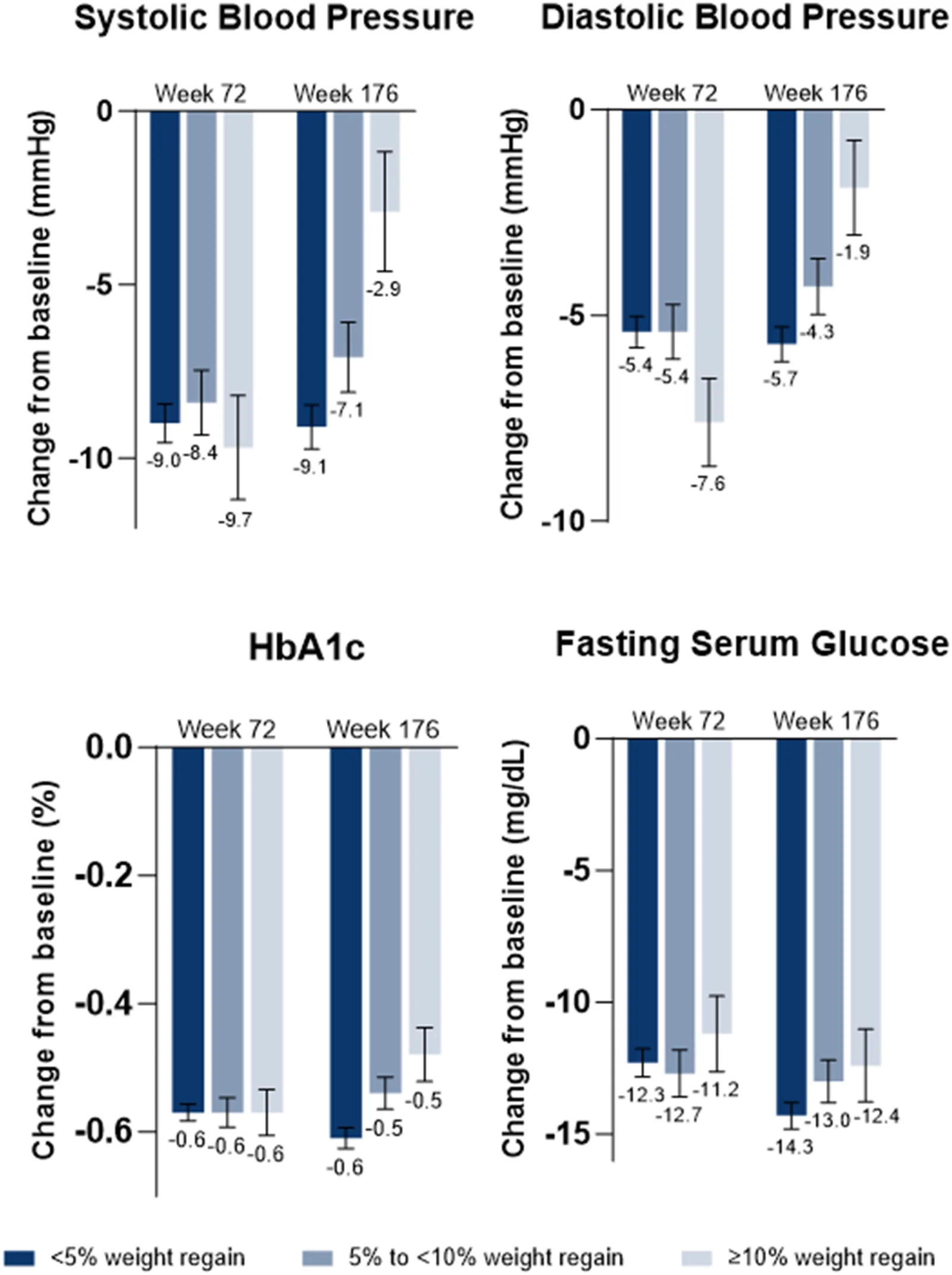
Change from baseline at Week 72 and Week 176 by level of weight regain for systolic blood pressure, diastolic blood pressure, HbA1c, and fasting serum glucose in participants receiving tirzepatide pooled 5 mg, 10 mg, 15 mg. Data are MBE (SE). Statistical analyses were performed using MMRM with model terms of baseline, country, sex, and treatment-by-time interaction. MMRM = mixed model for repeated measures; HbA1c = Hemoglobin A1c; MBE = model-based estimate; SE = standard error.

HbA1c was reduced from baseline across the weight regain groups and the reduction was sustained at Week 176 (Fig. 3 and Supplementary Table 5). The reduction was also seen across doses (Supplementary Tables 5, 6, and 7).

For lipid parameters, the mean improvements at Week 72 were sustained at week 176 across the weight regain groups and were consistent across doses (Fig. 4 and Supplementary Tables 7, 8, and 9).

**Fig. 4:**
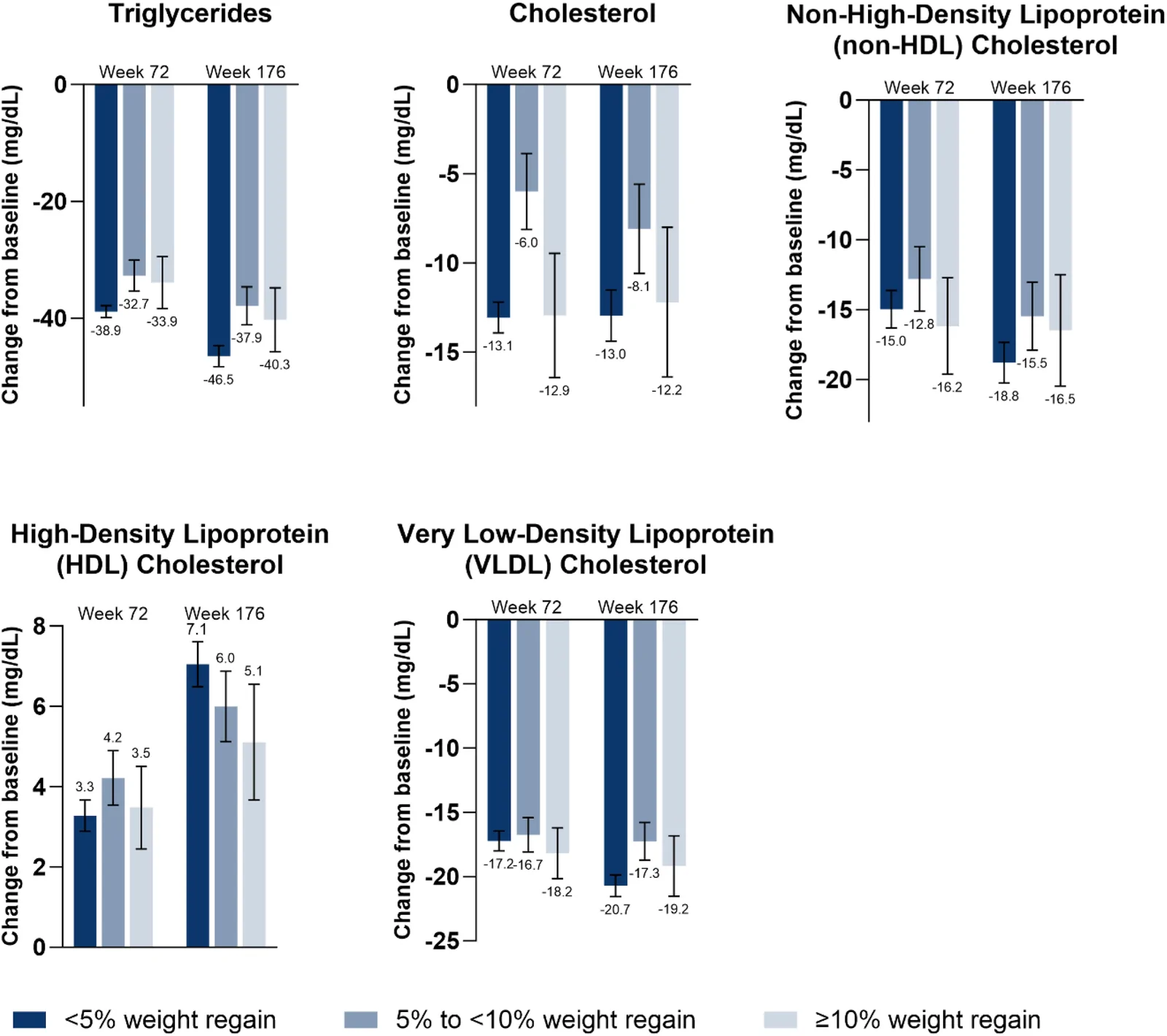
Change from baseline at Week 72 and Week 176 by level of weight regain for triglycerides, total cholesterol, non-HDL cholesterol, HDL cholesterol, VLDL cholesterol in participants receiving tirzepatide pooled 5 mg, 10 mg, 15 mg. Data are Estimate (SE). VLDL cholesterol was calculated based on triglycerides. Statistical analyses were performed using MMRM on log-transformed measurements with model terms of log-transformed baseline, country, sex, and treatment-by-time interaction, using Type III sum of squares. HDL = High-Density Lipoprotein; MMRM = mixed model for repeated measures; VLDL = Very Low-Density Lipoprotein; SE = standard error.

### 3.5 Weight regain from nadir and normoglycemia, prediabetes, and type 2 diabetes

At Week 176 normoglycemia occurred in 96.1%, 92.6%, and 91.2% in the <5%, 5% to <10%, ≥10% weight regain groups (Table 2), respectively. Concordantly, 2.9%, 5.4%, and 8.8% had prediabetes, respectively. Type 2 diabetes (T2D) was confirmed in 5 (1.0%), 3 (2.0%), and 0 (0%) participants in the <5%, 5% to <10%, and ≥10% weight regain groups, respectively.

## 4 Discussion

This post hoc analysis evaluated how weight regain from weight nadir was associated with parameters predicting cardiometabolic risk in SURMOUNT-1 3-year study of participants with obesity or overweight and prediabetes treated with tirzepatide for over three years. Nearly 70% of participants had weight increases of <5% of their baseline body weight from time of nadir, and 8% regained ≥10%. At Week 176, the percent of weight reduction maintained from time of weight nadir was 94.8%, 69.9% and 44.3%, for the 3 weight regain groups, respectively. By study end, 87% of studied participants either maintained or improved their WHtR category from time of weight nadir, including 94% among the group regaining the least weight. At baseline, 93% of all participants were classified within the highest risk WHtR category. At the time of weight nadir, WHtR reductions were comparable across the three weight-regain groups, with the proportion decreasing to approximately 43% of participants maintaining a WHtR >0.59. However, by the end of the study, there was a graded association for WHtR risk category across the weight regain groups (Fig. 2B). While the end of study prevalence of high risk WHtR did show a relationship with weight regain, over 90% of participants in each weight regain group were normoglycemic at Week 176, demonstrating that weight regain was not associated with an impact on achieving normoglycemia. This apparent dissociation between worsening central adiposity and preserved glycemic benefit during continued treatment may reflect a direct pharmacological effect of tirzepatide on β-cell function and insulin sensitivity beyond those attributable to weight reduction alone. Consistent with this, in a post hoc analyses of SURMOUNT-1 participants with obesity/overweight, without diabetes, treated with tirzepatide for 72 weeks, demonstrated that tirzepatide-associated improvements in β-cell function were independent of weight loss, while insulin sensitization was partly driven by weight-independent glucose-dependent insulinotropic polypeptide (GIP) receptor activation [13].

WHtR was used as the primary measure of central adiposity in this analysis, given that WC was collected at all time points coinciding with body weight measurement, allowing direct pairing with weight nadir. WHtR is a well established, practical anthropometric index that accounts for height when estimating central adiposity, offering advantages over waist circumference alone and BMI in predicting cardiometabolic risk [14]. At baseline, 94% of participants had WHtR >0.59, indicating the highest health risk category. At the time of weight nadir WHtR had decreased, indicating a downward shift in predicted health risk category, and was similar in the 3 weight groups. Further, from the time of weight nadir to Week 176, 87% sustained or improved their WHtR category: 94% of the 70% with <5% weight regain, 76% of the 22% with 5-<10% weight regain, and 63% of the 8% with ≥10% weight regain. WHtR category shifts from time of weight nadir to Week 176 provide a clinically meaningful lens through which to evaluate whether weight regain from nadir translates into meaningful worsening of central adiposity and associated cardiometabolic risk burden [14].

In this study, cardiometabolic parameters (SBP, DBP, lipids, and glycemia-related parameters) were associated with improvements from baseline to weight nadir, and these improvements were sustained across weight regain groups except for SBP and DBP in those treated with tirzepatide 10 mg who had weight regain ≥10% and for fasting glucose in those treated with 15 mg tirzepatide who had weight regain ≥10%. The change in fasting glucose increased from −13.3 (2.93) mg/dL to −10.8 (2.44) mg/dL in those participants. In the other weight regain and treatment groups, fasting glucose was reduced up to −13.5 (1.00) mg/dL and −14.8 (1.76) mg/dL at Week 72 and up to −14.9 (0.75) mg/dL and −14.3 (1.27) mg/dL at Week 176 for the <5% and 5% - <10% weight regain group, respectively. A recent post hoc analysis of SURMOUNT-4 evaluated participants who lost at least 10% of their body weight during the open-label tirzepatide phase (weeks 0–36) and were then switched to placebo. These participants were divided into four groups based on the percent of body weight regained by Week 88 after the open-label weight-loss period: less than 25%, 25–49%, 50–74%, and 75% or more (Supplementary Table 10). Contrary to the findings of our study, after withdrawal of tirzepatide, from week 36 to Week 88, the mean change in SBP increased by weight regain category to (6.8 mmHg [95% CI, 3.9–9.7], 7.3 mmHg [95% CI, 4.8–9.8], 9.6 mmHg [95% CI, 7.1–12.1], and 10.4 mmHg [95% CI, 8.0–12.8], respectively; *P* = 0.002), non-High-Density Lipoprotein (HDL) (−0.4% [95% CI, −7.3 to 6.5], 1.6% [95% CI, −2.3 to 5.5], 8.4% [95% CI, 3.9–12.9], and 10.8% [95% CI, 5.3–16.3], respectively), HbA_1c_ (0.14% [95% CI, 0.06–0.22], 0.15% [95% CI, 0.09–0.21], 0.27% [95% CI, 0.21–0.33], and 0.35% [95% CI, 0.29–0.41], respectively; *P* < 0.001) and fasting serum glucose (3.8 mg/dL [95% CI, 1.6–6.0], 6.9 mg/dL [95% CI, 5.1–8.7], 9.2 mg/dL [95% CI, 7.0–11.4], and 9.0 mg/dL [95% CI, 7.0–11.0], respectively; *P* < 0.001). Indirectly comparing the findings from our current analyses and the SURMOUNT-4 randomized withdrawal of tirzepatide, a consistent concept is that groups with greater weight regain experienced greater attenuation of cardiometabolic benefit. However, the magnitude of this association was substantially greater in the post-withdrawal setting. Continued tirzepatide treatment appears to be associated with preservation of cardiometabolic benefits, particularly for glycemia, lipids, and blood pressure, even in the presence of moderate weight regain, suggesting an active pharmacological contribution beyond weight mediation. In contrast, post-withdrawal in SURMOUNT-4, even limited weight regain, as little as approximately 2–3 kg (representing less than 25% of the weight lost during treatment) (Supplementary Table 10), was associated with a clinically meaningful increase in systolic blood pressure of 6.8 mmHg. Greater weight regain (approximately ≥75% of their initial weight loss) was associated with reversal of cardiometabolic parameters largely to pretreatment baseline values. These findings may have important implications for clinical practice and patient counseling.

By Week 176, participants with ≥10% weight regain had the highest proportion with worsening WHtR category from weight nadir and showed attenuation of earlier blood pressure benefits. This indicates that, as with other chronic diseases, some adults with obesity who are adherent to highly effective treatments may need multiple therapies to maintain goals. While some earlier cardiometabolic parameter benefits were lessened by the study's end, all parameters remained improved from baseline in all weight regain groups. Understanding changes in long-term treatment effect has shaped the management of many diseases, with secondary failure in T2D being one of the more well established concepts. This failure is driven by progressive β-cell dysfunction, not loss of drug potency [6]. In the current analysis of over 3 years of treatment with tirzepatide, 8% of participants experienced ≥10% weight regain calculated from pretreatment weight, which appears to be clinically relevant. Future research into the mechanisms of variability in weight changes during OM treatment may help develop new therapeutics and dispel misconceptions that patient choices are the main cause.

Metabolic bariatric surgery is well established as a highly effective treatment for advanced obesity. However, weight regain from the lowest post-operative weight is almost universal: 47.8% of people regain at least 10% of their maximum weight lost within a year of reaching nadir weight, rising to 86.5% by five years [15]. Hypothetically, this post-operative nadir may represent a physiological “undershoot”, with partial regain reflecting a return to a new equilibrium. Regain of ≥10% of presurgical weight after surgery has been reported in 8% at one year and 37% at three years [15]. In this study, the categories for percent weight regain from nadir were similarly calculated from pretreatment baseline. Weight regain impacts cardiometabolic benefits and can carry psychological burdens, including negative emotions and self-blame. Patient education may help alleviate this. In SURMOUNT-4, those switched from tirzepatide to placebo regained significant weight and experienced diminished quality of life compared to those who continued tirzepatide [16]. More research is needed to understand how weight regain during OM treatment affects psychological health. The current work aims to inform clinical discussions on weight change during treatment and the clinical relevance of weight regain regarding cardiometabolic health, supporting a focus on long-term well-being even when weight regain occurs.

The Swedish Obese Subjects (SOS) Study, which prospectively followed ∼ 2000 people who underwent bariatric surgery and ∼2000 contemporaneously matched controls with obesity, provides compelling evidence that the preservation of weight nadir does not dictate benefit. Peak weight reduction was achieved at 1–2 years post-operatively across all three surgical modalities, reaching a maximum of 32% from baseline for gastric bypass, 25% for vertical banded gastroplasty, and 20% for banding. Progressive weight regain occurred, with weight reduction by 10 years being 25%, 16%, and 14%, respectively. Despite this partial weight regain, the SOS study reported a landmark reduction in all-cause mortality at a mean follow-up of 10.9 years, with an adjusted overall mortality hazard ratio of 0.71 (95% CI 0.54–0.92, p = 0.01) in favor of surgery [17]. At a median follow-up of 24 years, bariatric surgery was associated with an adjusted median gain in life expectancy of 3.0 years compared with controls, driven by significant reductions in both cardiovascular death and cancer mortality [18].

## 5 Limitations

Several limitations of the present study should be acknowledged. WHtR was the only cardiometabolic risk parameter available at the three primary time points, baseline, time of weight nadir, and Week 176. The other cardiometabolic risk parameters were measured at baseline, Week 72, and Week 176. The interpretation of the association between weight regain from time of weight nadir to Week 176 and these other cardiometabolic risks is limited, as ∼55% of participants reached their weight nadir between Week 72 and Week 176. The one caveat to this is that when reviewing the change over time curves for the cardiometabolic risk parameters from the primary SURMOUNT-1 3-year study disclosure, the majority of the decrease in SBP, HbA1c, and glucose occur many weeks before Week 72. All data interpretations should include that the current analyses included participants who had at least 5% weight reduction by weight nadir and were 75% compliant with treatment. Other limitations include the post hoc nature of the study and the sample size, especially in the ≥10% weight regain group, and only participants with prediabetes were included in the SURMOUNT-1 3-year study, therefore, this analysis.

## 6 Conclusion

This current study demonstrates that during ongoing treatment with tirzepatide, the vast majority of participants sustained their weight reduction and early cardiometabolic benefits in WHtR, blood pressure, glycemia and lipids for over three years. Over 90% were normoglycemic at 176 weeks, despite having prediabetes at baseline, irrespective of degree of weight regain. Only 8% of participants experienced a weight increase of 10% (calculated from their pretreatment weight) or more after their weight nadir. While benefits in lipids and glycemia were sustained despite a 10% or more weight increase, there was an attenuation of the earlier benefits in WHtR and blood pressure at the end of study. Estimated means of all cardiometabolic risk parameters were improved at the end of study compared to pretreatment in all three weight regain groups. These data aim to allow a more informed clinical conversation to the relevance of weight nadir during long-term treatment with tirzepatide and hopefully support focus on health benefits rather than absolute weight loss. Ideally, this work will also support future research to the mechanisms of weight regain during active treatment while dispelling the misconception that individual behavior is the cause.

**Key takeaway clinical messages**

• Weight fluctuations are expected during long-term obesity management. Although everyone reaches a weight nadir, some people may experience concern even when weight regain is limited.
• In this study of participants with prediabetes, the vast majority experienced stable weight after their weight nadir during more than 3 years of tirzepatide treatment as they sustained on average 70–95% of their maximum weight loss. This limited regain did not appear clinically meaningful, since improvements in cardiometabolic risk parameters were largely maintained.
• Regaining 10% or more of body weight from baseline was associated with some attenuation of earlier cardiometabolic benefits. These participants made up 8% of those treated with tirzepatide; at the end of the study, they sustained 44% of their maximum weight loss and 91% were normoglycemic. The high rate of sustained normoglycemia may reflect direct tirzepatide effects.

## Credit author statement

GKD, JFB, and JD contributed to the conceptualization and design of the work. LJA, IJ, DC, and JD contributed to the acquisition of data and analysis of data. LJA, RPV, KRK, IJ, DC, GKD, and JD contributed to the interpretation of data. GKD, JFB, and JD contributed to writing the original draft. All authors contributed to critical revisions of the work.

## Ethical review

The phase 3, double-blind, randomized, placebo-controlled SURMOUNT-1 trial was conducted in accordance with the principles of the Declaration of Helsinki and the Good Clinical Practice guidelines and was approved by an independent ethics committee or institutional review board at each trial site. Written informed consent was obtained from all the participants. The ClinicalTrials.gov identifier for the studies reported here is NCT04184622 [11]. The list of Institutional Review Boards, which approved the trial has been previously reported in Krumholz et al., 2024 [19].

## Disclosure

L.J.A. reports receiving grants or personal fees from Altimmune, Amgen, Atria, Boehringer-Ingelheim, Carmot Therapeutics/Roche, CinFina Pharma, Corteria, Currax Pharma, Eli Lilly and Company, Enterin, Helicore Biopharma, Jamieson Wellness, Juvena Therapeutics, Kallyope, MBX Bioscience, Metsera, Novo Nordisk, Pfizer, Prosciento, Senda Biosciences, Skye Bio, Syntis Bio, Verdiva, Veru Pharmaceuticals and Zealand Pharmaceuticals; receiving institutional research funding from Eli Lilly, Novo Nordisk, Skye Bioscience and Viking Therapeutics; having equity interests in Clic Bio, ERX Pharmaceuticals, Flyte Health, Jamieson Wellness, Juvena Therapeutics, Kallyope, MBX Bioscience, Mediflix, Metsera, Skye Bio, Syntis Bio, Verdiva and Veru Pharmaceuticals; and serving on a board of directors for ERX Pharmaceuticals, Flyte Health and Jamieson Wellness.

R.P.V. reports grants from Recordati, Novo Nordisk and Novartis Pharma AG and honoraria from Novo Nordisk, Pfizer, Amgen, AstraZeneka and Amarin.

K.R.K. reports advisory/consulting fees support from Antag Therapeutics, Metsera, Novo Nordisk, Roche Pharmaceuticals, and vTv therapeutics; She has also received research-related contracts (paid to the institution) from Bayer, Boehringer Ingelheim, Carmot, Diasome, Eli Lilly, Gentibio, Novo Nordisk, Rhythm Pharmaceuticals, Roche Pharmaceuticals, and vTv Therapeutics.

J.F.B., I.J., D.C., G.K.D., and J.P.D. are employees and shareholders of Eli Lilly and Company.

## Data sharing statement

Lilly provides access to all individual participant data collected during the trial, after anonymization, with the exception of pharmacokinetic or genetic data. Data are available to request 6 months after the indication studied has been approved in the US and EU and after primary publication acceptance, whichever is later. No expiration date of data requests is currently set once data are made available. Access is provided after a proposal has been approved by an independent review committee identified for this purpose and after receipt of a signed data sharing agreement. Data and documents, including the study protocol, statistical analysis plan, clinical study report, blank or annotated case report forms, will be provided in a secure data sharing environment. For details on submitting a request, see the instructions provided at www.vivli.org.

## Declaration of artificial intelligence (AI) and AI-assisted technologies

During the preparation of this work the authors did not use AI or AI-assisted technologies.

## Source of funding

This work was funded by 10.13039/100004312Eli Lilly and Company, whose employees were involved in the design and conduct of the study; collection, management, analysis, and interpretation of the data; preparation, review, or approval of the manuscript; and decision to submit the manuscript for publication.
