# Supplementary material for: Evaluation of clinical relevance of weight nadirs and weight regain during 3 years of treatment with tirzepatide in participants with obesity or overweight and prediabetes
Source: Obes Pillars. 2026 Sep 18;20:100333. doi: 10.1016/j.obpill.2026.100333 (PMC13634289; doi:10.1016/j.obpill.2026.100333)
Supplement: Supplementary file 1 [file mmc1.pdf]

# Supplementary appendix

**Supplement to: Aronne et al. Evaluation of clinical relevance of weight nadirs and weight regain during 3 years of treatment with tirzepatide in participants with obesity or overweight and prediabetes**

**Table of Contents**

Supplementary figure 1 .................... 3
Supplementary figure 2 .................... 4
Supplementary figure 3. .................... 6
Supplementary table 1 .................... 7
Supplementary table 2 .................... 8
Supplementary table 3 .................... 9
Supplementary table 4 .................... 10
Supplementary table 5 .................... 11
Supplementary table 6 .................... 13
Supplementary table 7 .................... 14
Supplementary table 8 .................... 16
Supplementary table 9 .................... 18
Supplementary table 10 .................... 19

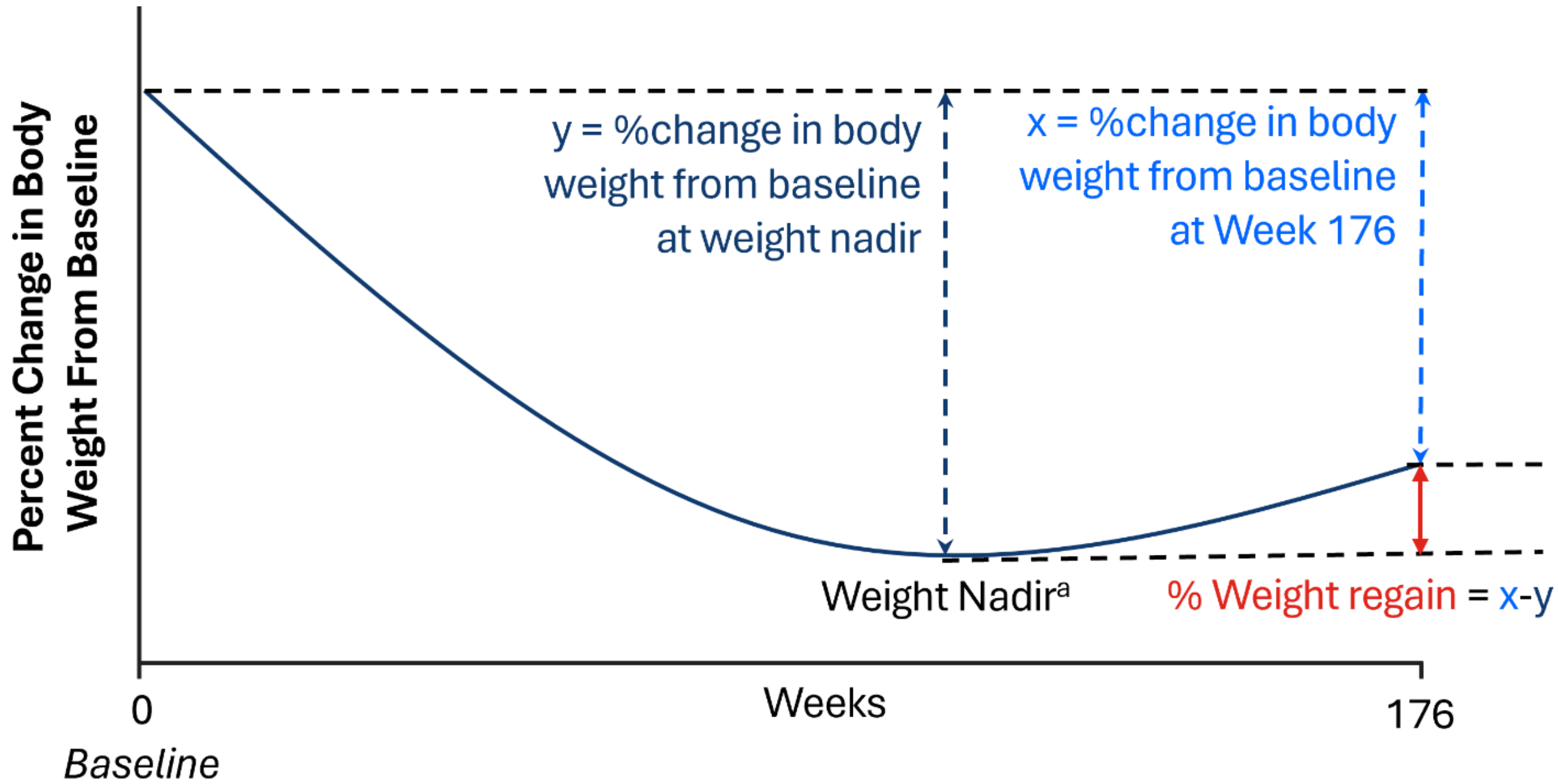


**Supplementary figure 1.** Change in body weight at various timepoints relative to weight nadir. Weight nadir was defined as on-treatment weight with maximum percent weight reduction from baseline (first one if weight nadir was observed at multiple visits) (ie, the lowest weight achieved during treatment). Percent weight regain from weight nadir refers to percent of baseline (pretreatment) body weight. W=Week.

A

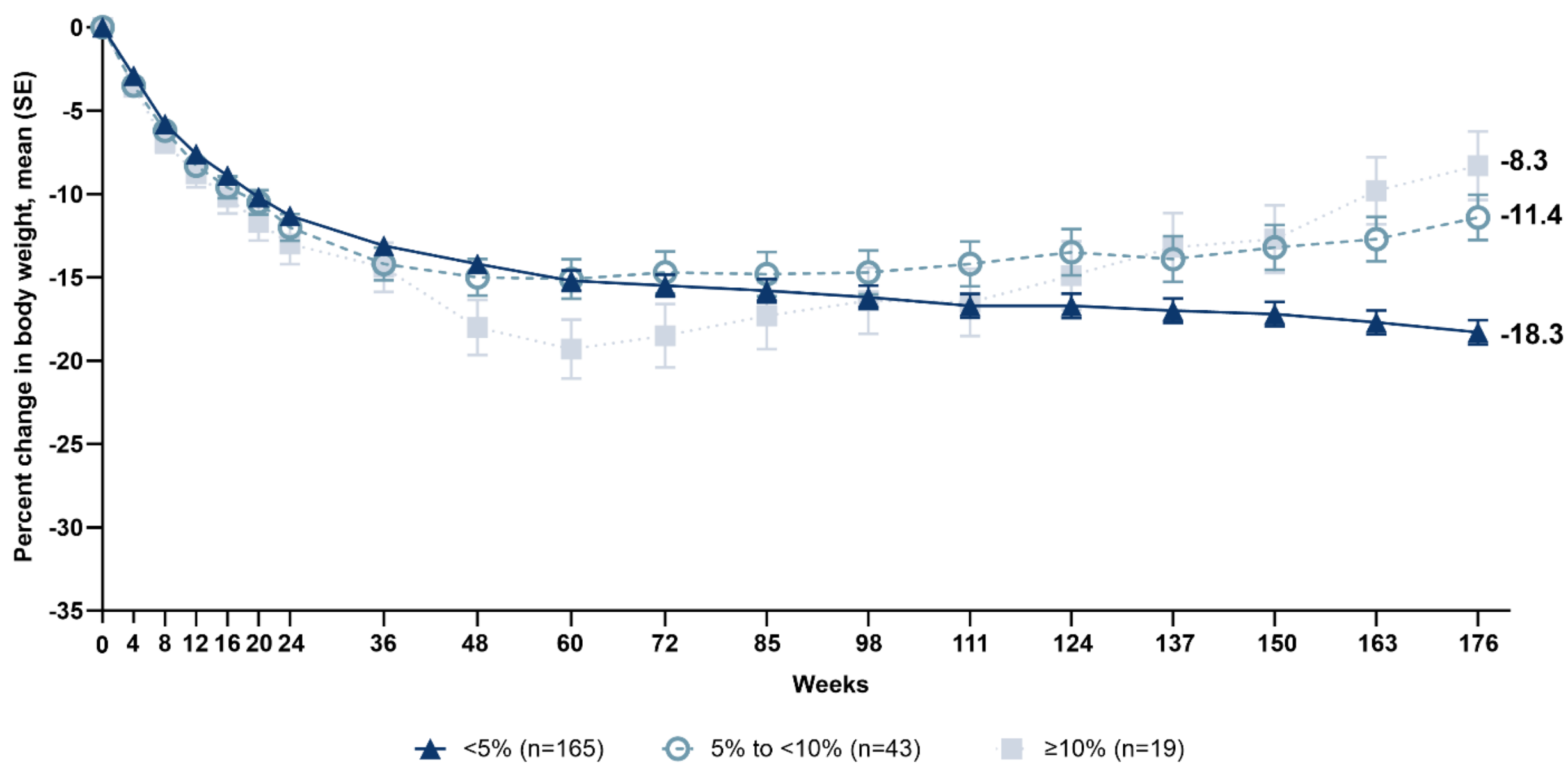


B

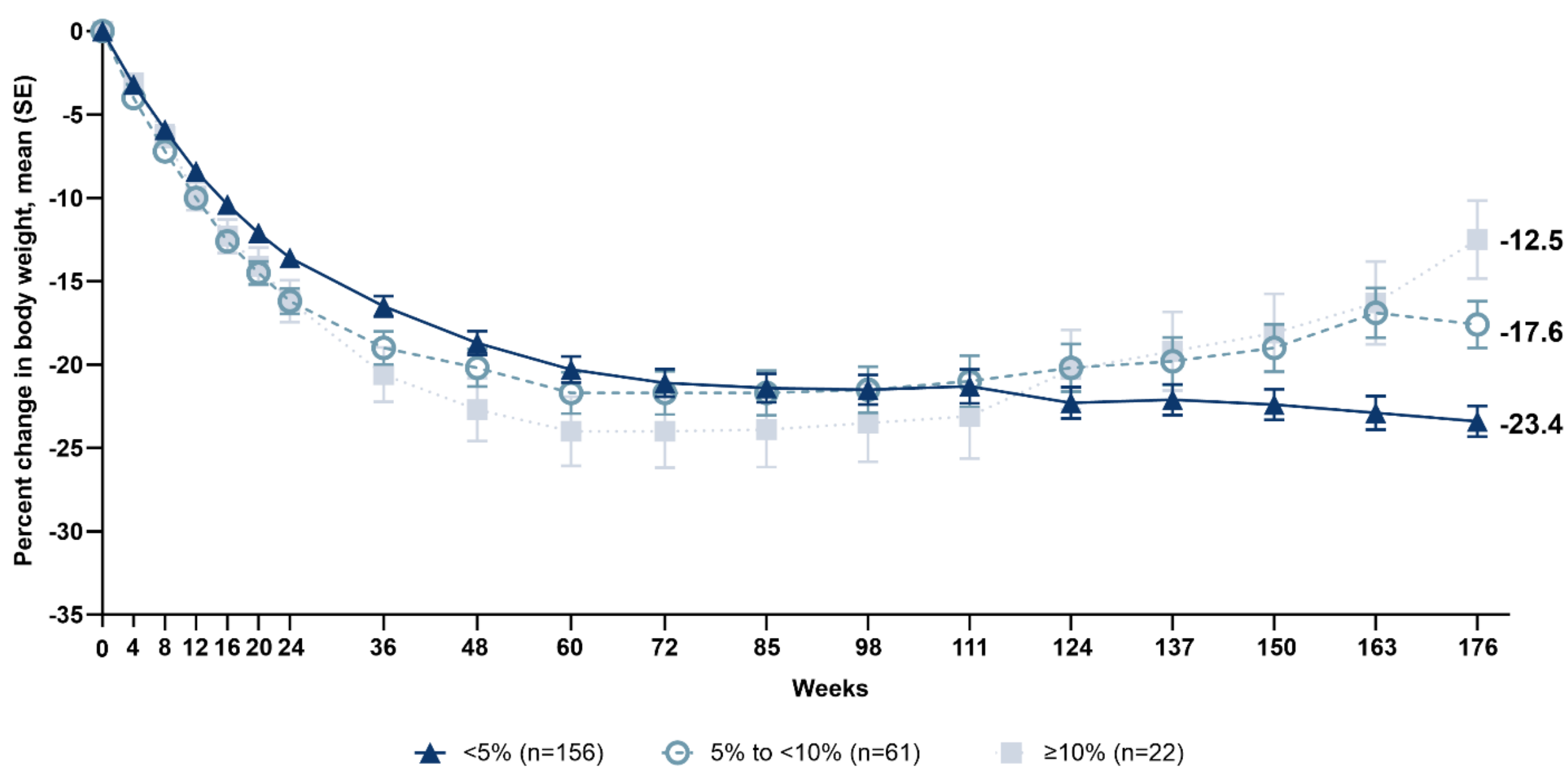


**Supplementary figure 2.** Percent change in body weight by visit by level of weight regain from baseline to Week 176 in participants receiving **A.** tirzepatide 5 mg and **B.** 10 mg by category of weight loss. Data are MBE (SE). Statistical analyses were performed using MMRM with model terms of baseline, country, sex, time, treatment, and treatment-by-time interaction, using Type III

sum of squares. MMRM=mixed model for repeated measures; MBE=model-based estimate; SE=standard error.

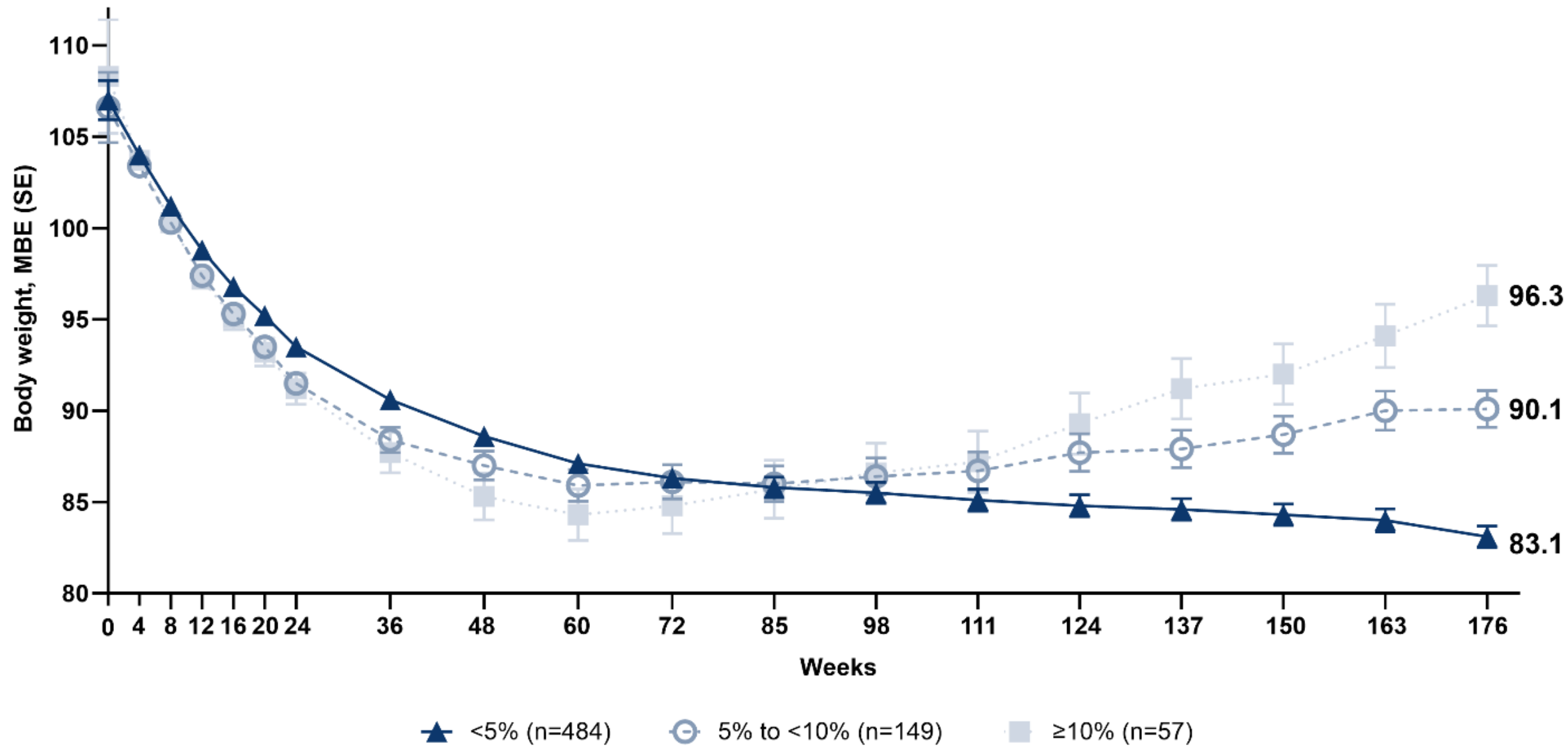


**Supplementary figure 3.** Body weight (kg) by visit by weight regain group from baseline to Week 176 in participants receiving tirzepatide pooled 5 mg, 10 mg, 15 mg. Data are MBE (SE). Statistical analyses were performed using MMRM with model terms of baseline, country, sex, and treatment-by-time interaction. MMRM=mixed model for repeated measures; MBE=model-based estimate; SE=standard error.

**Supplementary table 1. Week 72 and Week 176 characteristics**

| Regain from weight nadir; % calculated from baseline weight | Sample size | Mean (SD) of BL BMI | Mean (SD) of Week 72 BMI | Mean (SD) of Week 176 BMI | % Compliance Week 0-72 | % Compliance Week 73-176 | % Early Treatment Discontinuation Week 0-72 | % Early Treatment Discontinuation Week 73-176 |
|---|---|---|---|---|---|---|---|---|
| <5% | 484 | 38.6 (7.09) | 31.0 (7.05) | 29.7 (6.58) | 98.3 | 98.5 | 18.6 (90 out of 484) | 10.1 (49 out of 484) |
| 5-<10% | 149 | 38.3 (6.89) | 30.6 (7.13) | 31.2 (6.12) | 98.9 | 98.4 | 1.3 (2 out of 149) | 5.4 (8 out of 149) |
| ≥10% | 57 | 39.8 (6.37) | 31.4 (6.52) | 34.7 (6.50) | 98.2 | 98.4 | 0 (0 out of 57) | 10.5 (6 out of 57) |

Data are mean (SD) unless otherwise noted. BL=baseline

**Supplementary table 2. Additional Demographic and Clinical Characteristics of the Participants at Baseline.**

| Characteristic | % Weight Regain from Baseline <5% (N=484) | % Weight Regain from Baseline 5-<10% (N=149) | % Weight Regain from Baseline ≥10% (N=57) | Total (n=690) |
|---|---|---|---|---|
| Height, cm | 166.3 (9.5) | 166.4 (10.4) | 164.6 (9.4) | 166.2 (9.7) |
| Duration of Obesity, years | 15.6 (11.4) | 15.5 (10.4) | 13.8 (10.3) | 15.5 (11.1) |
| Lipid levels, mean mg/dl | | | | |
| Cholesterol | 189.5 (38.2) | 192.5 (37.1) | 196.0 (36.7) | 190.7 (37.8) |
| HDL Cholesterol | 48.5 (12.5) | 48.2 (12.5) | 47.4 (9.4) | 48.3 (12.3) |
| Non-HDL Cholesterol | 141.0 (36.1) | 144.3 (35.8) | 148.7 (35.5) | 142.3 (36.0) |
| LDL Cholesterol | 111.1 (32.4) | 115.0 (32.7) | 120.8 (32.6) | 112.7 (32.6) |
| VLDL Cholesterol | 67.7 (31.3) | 66.8 (28.8) | 63.6 (22.1) | 67.2 (30.1) |
| Triglycerides | 150.4 (75.0) | 156.6 (107.0) | 139.0 (48.2) | 150.8 (81.3) |
| Hypertension, n (%) | 206 (42.6) | 65 (43.6) | 16 (28.1) | 287 (41.6) |
| Obstructive sleep apnea, n (%) | 17 (3.5) | 10 (6.7) | 5 (8.8) | 32 (4.6) |
| Dyslipidemia, n (%) | 177 (36.6) | 45 (30.2) | 16 (28.1) | 238 (34.5) |
| Atherosclerotic CVD, n (%) | 25 (5.2) | 4 (2.7) | 2 (3.5) | 31 (4.5) |
| Number of weight-related complications, n (%)[&] | | | | |
| ≥2 | 211 (43.6) | 62 (41.6) | 18 (31.6) | 291 (42.2) |
| 1 | 134 (27.7) | 44 (29.5) | 10 (17.5) | 188 (27.2) |
| 0 | 139 (28.7) | 43 (28.9) | 29 (50.9) | 211 (30.6) |

Data are mean (SD) unless otherwise noted.

† Race or ethnic group was reported by the participant& Weight-related complications excluding study diagnosed prediabetes, which was present in all participants included in the present post hoc analysis.

HbA1c=Hemoglobin A1c; Atherosclerotic CVD = Atherosclerotic cardiovascular disease; HDL = High-Density Lipoprotein; LDL = Low-Density Lipoprotein; VLDL = Very Low-Density Lipoprotein;

**Supplementary table 3.** Change in BMI at Week 72 and Week 176 in participants receiving tirzepatide 5 mg, 10 mg, 15 mg by category of weight loss.

| | **Tirzepatide 5 mg** | | | **Tirzepatide 10 mg** | | | **Tirzepatide 15 mg** | | |
|---|---|---|---|---|---|---|---|---|---|
| MBE (SE), kg/m$^2$ | **<5% weight regain** | **5% to <10% weight regain** | **≥10% weight regain** | **<5% weight regain** | **5% to <10% weight regain** | **≥10% weight regain** | **<5% weight regain** | **5% to <10% weight regain** | **≥10% weight regain** |
| Week 72 | -5.8 (0.26) | -5.6 (0.50) | -7.3 (0.75) | -8.3 (0.36) | -8.4 (0.56) | -9.6 (0.93) | -9.0 (0.33) | -8.9 (0.61) | -8.0 (1.02) |
| Week 176 | -6.8 (0.29) | -4.4 (0.55) | -3.3 (0.83) | -9.2 (0.38) | -6.8 (0.59) | -5.2 (0.98) | -10.3 (0.37) | -7.5 (0.67) | -4.0 (1.16) |

Data are MBE (SE). Statistical analyses were performed using MMRM with model terms of baseline, country, sex, time, treatment, and treatment-by-time interaction, using Type III sum of squares. BMI = Body mass index; MBE= model-based estimate; SE=standard error.

**Supplementary table 4**. Percent change in body weight by visit from weight nadir to Week 176 in participants receiving tirzepatide pooled 5 mg, 10 mg, 15 mg by category of weight loss.

| | **Pooled tirzepatide 5 mg, 10 mg, 15 mg** | | |
|---|---|---|---|
| MBE (SE) | **<5% weight regain** | **5% to <10% weight regain** | **≥10% weight regain** |
| Week 4 | -3.1 (0.09) | -3.8 (0.17) | -3.4 (0.27) |
| Week 8 | -5.8 (0.14) | -6.8 (0.25) | -6.6 (0.39) |
| Week 12 | -8.1 (0.17) | -9.5 (0.31) | -9.5 (0.51) |
| Week 16 | -10.0 (0.21) | -11.6 (0.38) | -11.5 (0.61) |
| Week 20 | -11.5 (0.24) | -13.3 (0.43) | -13.3 (0.70) |
| Week 24 | -13.1 (0.27) | -15.2 (0.48) | -15.1 (0.77) |
| Week 36 | -15.8 (0.34) | -18.1 (0.61) | -18.3 (0.98) |
| Week 48 | -17.7 (0.39) | -19.4 (0.69) | -20.7 (1.12) |
| Week 60 | -19.1 (0.43) | -20.4 (0.76) | -21.6 (1.23) |
| Week 72 | -19.9 (0.46) | -20.2 (0.82) | -21.2 (1.32) |
| Week 85 | -20.3 (0.49) | -20.4 (0.85) | -20.3 (1.38) |
| Week 98 | -20.6 (0.50) | -20.0 (0.87) | -19.5 (1.40) |
| Week 111 | -20.9 (0.53) | -19.7 (0.90) | -19.0 (1.46) |
| Week 124 | -21.2 (0.51) | -18.8 (0.89) | -16.8 (1.44) |
| Week 137 | -21.4 (0.51) | -18.5 (0.88) | -15.2 (1.42) |
| Week 150 | -21.6 (0.51) | -17.8 (0.88) | -14.4 (1.42) |
| Week 163 | -21.8 (0.54) | -16.6 (0.91) | -12.5 (1.49) |
| Week 176 | -22.6 (0.50) | -16.5 (0.86) | -10.2 (1.40) |

Data are MBE (SE). Statistical analyses were performed using MMRM with model terms of baseline, country, sex, and treatment-by-time interaction. MMRM=mixed model for repeated measures; MBE=model-based estimate; SE=standard error.

**Supplementary table 5.** Systolic blood pressure, diastolic blood pressure, HbA1c, and fasting serum glucose at baseline, Week 72, and Week 176 in participants receiving tirzepatide 5 mg, 10 mg, 15 mg by category of weight loss.

| | **Tirzepatide 5 mg** | | | **Tirzepatide 10 mg** | | | **Tirzepatide 15 mg** | | |
|---|---|---|---|---|---|---|---|---|---|
| MBE (SE) | **<5% weight regain** | **5% to <10% weight regain** | **≥10% weight regain** | **<5% weight regain** | **5% to <10% weight regain** | **≥10% weight regain** | **<5% weight regain** | **5% to <10% weight regain** | **≥10% weight regain** |
| Systolic Blood Pressure, mmHg | | | | | | | | | |
| Week 0 | 126.9 (0.96) | 127.5 (1.87) | 127.8 (2.81) | 126.2 (1.06) | 125.4 (1.69) | 121.2 (2.82) | 125.5 (0.98) | 126.9 (1.86) | 125.3 (3.12) |
| Week 72 | 117.3 (0.90) | 121.0 (1.68) | 116.7 (2.52) | 117.1 (0.96) | 115.2 (1.45) | 119.6 (2.40) | 117.6 (0.97) | 118.1 (1.77) | 111.4 (2.95) |
| Week 176 | 117.8 (1.07) | 123.7 (1.92) | 126.3 (3.01) | 118.6 (1.13) | 117.3 (1.57) | 124.2 (2.64) | 116.0 (1.12) | 117.0 (1.90) | 117.6 (3.64) |
| | | | | | | | | | |
| Diastolic Blood Pressure, mmHg | | | | | | | | | |
| Week 0 | 80.6 (0.63) | 81.2 (1.24) | 81.7 (1.86) | 80.5 (0.70) | 80.7 (1.12) | 80.4 (1.86) | 80.2 (0.61) | 81.0 (1.16) | 80.0 (1.94) |
| Week 72 | 74.7 (0.61) | 76.9 (1.12) | 72.9 (1.68) | 74.5 (0.70) | 74.1 (1.05) | 74.0 (1.74) | 76.2 (0.69) | 74.7 (1.27) | 71.2 (2.12) |
| Week 176 | 75.0 (0.76) | 78.1 (1.36) | 78.7 (2.16) | 75.6 (0.78) | 76.8 (1.08) | 79.9 (1.82) | 74.2 (0.68) | 73.5 (1.16) | 75.0 (2.20) |
| | | | | | | | | | |
| HbA1c, % | | | | | | | | | |
| Week 0 | 5.8 (0.023) | 5.8 (0.045) | 5.7 (0.068) | 5.7 (0.027) | 5.8 (0.043) | 5.8 (0.071) | 5.8 (0.031) | 5.7 (0.06) | 5.6 (0.1) |
| Week 72 | 5.3 (0.021) | 5.3 (0.039) | 5.2 (0.059) | 5.2 (0.023) | 5.1 (0.035) | 5.2 (0.057) | 5.1 (0.023) | 5.1 (0.042) | 5.2 (0.069) |
| Week 176 | 5.2 (0.026) | 5.3 (0.046) | 5.3 (0.072) | 5.1 (0.028) | 5.2 (0.039) | 5.2 (0.066) | 5.1 (0.026) | 5.1 (0.044) | 5.3 (0.082) |
| | | | | | | | | | |
| Fasting Serum Glucose, mg/dL | | | | | | | | | |
| Week 0 | 102.0 (0.70) | 101.1 (1.37) | 99.5 (2.06) | 101.1 (0.81) | 102.5 (1.30) | 102.9 (2.16) | 101.8 (0.73) | 101.0 (1.39) | 98.4 (2.33) |
| Week 72 | 91.5 (0.76) | 94.3 (1.41) | 90.7 (2.13) | 88.5 (0.97) | 86.5 (1.42) | 92.1 (2.35) | 87.6 (1.00) | 86.4 (1.76) | 87.9 (2.93) |
| Week 176 | 89.0 (1.00) | 94.0 (1.79) | 88.7 (2.87) | 86.2 (0.83) | 86.0 (1.13) | 88.9 (1.89) | 86.3 (0.75) | 86.9 (1.27) | 90.4 (2.44) |

Data are MBE (SE). Statistical analyses were performed using MMRM with model terms of baseline, country, sex, time, treatment, and treatment-by-time interaction, using Type III sum of

squares. MMRM=mixed model for repeated measures; HbA1c=Hemoglobin A1c; MBE=model-based estimate; SE=standard error.

**Supplementary table 6.** Systolic blood pressure, diastolic blood pressure, HbA1c, and fasting serum glucose at baseline, Week 72, and Week 176 in participants receiving pooled tirzepatide 5 mg, 10 mg, 15 mg by category of weight loss.

| | **Pooled tirzepatide 5 mg, 10 mg, 15 mg** | | |
|---|---|---|---|
| MBE (SE) | **<5% weight regain** | **5% to <10% weight regain** | **≥10% weight regain** |
| Systolic Blood Pressure, mmHg | | | |
| Week 0 | 126.2 (0.58) | 126.5 (1.04) | 124.5 (1.68) |
| Week 72 | 117.3 (0.55) | 117.9 (0.94) | 116.6 (1.50) |
| Week 176 | 117.2 (0.63) | 119.2 (1.01) | 123.4 (1.72) |
| | | | |
| Diastolic Blood Pressure, mmHg | | | |
| Week 0 | 80.4 (0.37) | 80.9 (0.67) | 80.7 (1.09) |
| Week 72 | 75.1 (0.38) | 75.1 (0.66) | 72.9 (1.06) |
| Week 176 | 74.8 (0.42) | 76.2 (0.68) | 78.6 (1.15) |
| | | | |
| HbA1c, % | | | |
| Week 0 | 5.8 (0.016) | 5.8 (0.028) | 5.8 (0.046) |
| Week 72 | 5.2 (0.013) | 5.2 (0.023) | 5.2 (0.036) |
| Week 176 | 5.1 (0.016) | 5.2 (0.025) | 5.3 (0.042) |
| | | | |
| Fasting serum glucose, mg/dL | | | |
| Week 0 | 101.6 (0.43) | 101.6 (0.78) | 100.5 (1.26) |
| Week 72 | 89.2 (0.53) | 88.8 (0.89) | 90.3 (1.43) |
| Week 176 | 87.2 (0.50) | 88.5 (0.81) | 89.1 (1.38) |

Data are MBE (SE). Statistical analyses were performed using MMRM on log-transformed measurements with model terms of log-transformed baseline, country, sex, and treatment-by-time interaction. MMRM=mixed model for repeated measures; HbA1c=Hemoglobin A1c; MBE=model-based estimate; SE=standard error.

**Supplementary table 7.** Change from baseline for systolic blood pressure, diastolic blood pressure, HbA1c, fasting serum glucose, triglycerides, total cholesterol, non-HDL cholesterol, HDL cholesterol, and VLDL cholesterol at Week 72, and Week 176 in participants receiving tirzepatide 5 mg, 10 mg, 15 mg by category of weight loss.

| | **Tirzepatide 5 mg** | | | **Tirzepatide 10 mg** | | | **Tirzepatide 15 mg** | | |
|---|---|---|---|---|---|---|---|---|---|
| MBE (SE) | **<5% weight regain** | **5% to <10% weight regain** | **≥10% weight regain** | **<5% weight regain** | **5% to <10% weight regain** | **≥10% weight regain** | **<5% weight regain** | **5% to <10% weight regain** | **≥10% weight regain** |
| Systolic Blood Pressure, mmHg | | | | | | | | | |
| Week 72 | -9.9 (0.90) | -6.3 (1.68) | -10.6 (2.52) | -8.5 (0.96) | -10.4 (1.45) | -6.0 (2.40) | -8.4 (0.97) | -7.9 (1.77) | -14.6 (2.95) |
| Week 176 | -9.4 (1.07) | -3.6 (1.92) | -1.0 (3.01) | -7.0 (1.13) | -8.3 (1.57) | -1.5 (2.64) | -10.1 (1.12) | -9.0 (1.90) | -8.4 (3.64) |
| | | | | | | | | | |
| Diastolic Blood Pressure, mmHg | | | | | | | | | |
| Week 72 | -6.0 (0.61) | -3.7 (1.12) | -7.8 (1.68) | -6.1 (0.70) | -6.4 (1.05) | -6.5 (1.74) | -4.1 (0.69) | -5.6 (1.27) | -9.1 (2.12) |
| Week 176 | -5.7 (0.76) | -2.5 (1.36) | -2.0 (2.16) | -4.9 (0.78) | -3.8 (1.08) | -0.7 (1.82) | -6.1 (0.68) | -6.8 (1.16) | -5.3 (2.20) |
| | | | | | | | | | |
| HbA1c, % | | | | | | | | | |
| Week 72 | -0.5 (0.02) | -0.5 (0.04) | -0.6 (0.06) | -0.6 (0.02) | -0.6 (0.04) | -0.6 (0.06) | -0.6 (0.02) | -0.6 (0.04) | -0.6 (0.07) |
| Week 176 | -0.5 (0.03) | -0.5 (0.05) | -0.4 (0.07) | -0.6 (0.03) | -0.6 (0.04) | -0.5 (0.07) | -0.7 (0.03) | -0.6 (0.04) | -0.5 (0.08) |
| | | | | | | | | | |
| Fasting serum glucose, mg/dL | | | | | | | | | |
| Week 72 | -10.1 (0.76) | -7.3 (1.41) | -10.9 (2.13) | -13.2 (0.97) | -15.2 (1.42) | -9.6 (2.35) | -13.5 (1.00) | -14.8 (1.76) | -13.3 (2.93) |
| Week 176 | -12.6 (1.00) | -7.6 (1.79) | -12.9 (2.87) | -15.5 (0.83) | -15.7 (1.13) | -12.8 (1.89) | -14.9 (0.75) | -14.3 (1.27) | -10.8 (2.44) |
| | | | | | | | | | |
| Triglycerides, mg/dL | | | | | | | | | |
| Week 72 | -37.3 (1.93) | -23.7 (5.36) | -27.7 (8.76) | -34.6 (1.84) | -35.4 (4.04) | -32.9 (7.17) | -44.6 (1.63) | -37.3 (4.51) | -41.5 (7.23) |
| Week 176 | -39.4 (3.27) | -33.9 (6.22) | -43.5 (9.19) | -46.8 (3.39) | -36.2 (5.56) | -40.8 (8.93) | -52.5 (2.61) | -44.5 (5.17) | -35.8 (10.81) |
| | | | | | | | | | |
| Cholesterol, mg/dL | | | | | | | | | |
| Week 72 | -11.6 (1.49) | -0.8 (3.83) | -4.6 (6.36) | -12.2 (1.52) | -6.3 (3.46) | -12.5 (5.78) | -15.3 (1.47) | -11.2 (3.79) | -22.5 (5.94) |

| | | | | | | | | | |
|---|---|---|---|---|---|---|---|---|---|
| Week 176 | -13.0 (2.59) | -8.7 (4.90) | -7.4 (7.94) | -13.6 (2.55) | -7.6 (3.8) | -10.0 (6.35) | -12.1 (2.34) | -8.8 (4.47) | -21.1 (7.81) |
| | | | | | | | | | |
| Non-HDL cholesterol, mg/dL | | | | | | | | | |
| Week 72 | -14.9 (1.39) | -6.9 (3.63) | -8.4 (5.99) | -16.4 (1.46) | -10.8 (3.36) | -15.5 (5.59) | -19.6 (1.47) | -16.7 (3.80) | -24.4 (5.94) |
| Week 176 | -18.9 (2.40) | -13.1 (4.70) | -12.9 (7.60) | -21.4 (2.34) | -15.7 (3.59) | -17.0 (6.02) | -20.5 (2.11) | -14.4 (4.21) | -21.0 (7.44) |
| | | | | | | | | | |
| HDL cholesterol, mg/dL | | | | | | | | | |
| Week 72 | 3.4 (0.45) | 4.8 (1.13) | 2.9 (1.81) | 4.0 (0.46) | 4.3 (1.01) | 3.4 (1.71) | 3.8 (0.46) | 3.8 (1.16) | 1.5 (1.86) |
| Week 176 | 6.4 (0.86) | 4.8 (1.53) | 5.1 (2.49) | 7.1 (0.98) | 7.5 (1.47) | 6.8 (2.45) | 8.3 (0.89) | 5.1 (1.51) | -0.1 (2.60) |
| | | | | | | | | | |
| VLDL cholesterol*, mg/dL | | | | | | | | | |
| Week 72 | -16.7 (0.84) | -11.9 (2.30) | -12.9 (3.77) | -15.3 (0.84) | -15.4 (1.84) | -14.6 (3.24) | -20.2 (0.74) | -16.0 (2.09) | -21.0 (3.17) |
| Week 176 | -17.5 (1.44) | -14.0 (2.88) | -19.6 (4.09) | -21.2 (1.45) | -15.4 (2.4) | -17.9 (3.84) | -23.8 (1.18) | -19.6 (2.38) | -18.2 (4.69) |

*VLDL cholesterol was calculated based on triglycerides.

Data are MBE (SE). Statistical analyses for change from baseline for systolic blood pressure, diastolic blood pressure, HbA1c, and fasting serum glucose were performed using MMRM with model terms of baseline, country, sex, time, treatment, and treatment-by-time interaction. Statistical analyses for change from baseline for triglycerides, total cholesterol, non-HDL cholesterol, HDL cholesterol, and VLDL cholesterol were performed using MMRM on log-transformed measurements with model terms of log-transformed baseline, country, sex, time, treatment, and treatment-by-time interaction, using Type III sum of squares. MMRM=mixed model for repeated measures; HbA1c=Hemoglobin A; HDL = High-Density Lipoprotein; MBE=model-based estimate; LDL = Low-Density Lipoprotein; VLDL = Very Low-Density Lipoprotein; SE=standard error.

**Supplementary table 8.** Lipids at baseline, Week 72, and Week 176 in participants receiving pooled tirzepatide in participants receiving tirzepatide 5 mg, 10 mg, 15 mg by category of weight loss.

| | **Tirzepatide 5 mg** | | | **Tirzepatide 10 mg** | | | **Tirzepatide 15 mg** | | |
|---|---|---|---|---|---|---|---|---|---|
| MBE (SE) | **<5% weight regain** | **5% to <10% weight regain** | **≥10% weight regain** | **<5% weight regain** | **5% to <10% weight regain** | **≥10% weight regain** | **<5% weight regain** | **5% to <10% weight regain** | **≥10% weight regain** |
| Triglycerides, mg/dL | | | | | | | | | |
| Week 0 | 130.6 (2.94) | 129.9 (7.98) | 131.9 (13.51) | 126. 8 (2.89) | 120.4 (6.78) | 136.0 (13.82) | 126.1 (2.56) | 140.1 (8.05) | 149.7 (15.16) |
| Week 72 | 95.2 (1.93) | 108.8 (5.37) | 104.8 (8.77) | 93.8 (1.86) | 93.1 (4.09) | 95.6 (7.27) | 86.4 (1.63) | 93.8 (4.52) | 89.6 (7.23) |
| Week 176 | 93.8 (3.21) | 99.2 (6.11) | 89.8 (9.04) | 85.2 (3.17) | 95.0 (5.20) | 90.8 (8.34) | 80.3 (2.53) | 88.1 (5.00) | 96.5 (10.45) |
| | | | | | | | | | |
| Cholesterol, mg/dL | | | | | | | | | |
| Week 0 | 187.2 (1.80) | 183.7 (4.81) | 197.8 (8.64) | 190.3 (1.75) | 188.5 (4.28) | 187.8 (7.69) | 186.3 (1.69) | 192.2 (4.95) | 194.5 (8.82) |
| Week 72 | 175.8 (1.49) | 186.6 (3.83) | 182.9 (6.36) | 176.9 (1.51), | 182.7 (3.45) | 176.5 (5.77) | 171.9 (1.46) | 175.9 (3.77) | 164.7 (5.90) |
| Week 176 | 174.4 (2.58) | 178.8 (4.89) | 180.0 (7.93) | 175.3 (2.58), | 181.3 (3.83) | 178.9 (6.41) | 174.9 (2.36) | 178.2 (4.52) | 165.8 (7.89) |
| | | | | | | | | | |
| Non-HDL cholesterol, mg/dL | | | | | | | | | |
| Week 0 | 137.0 (1.67) | 135.5 (4.51) | 148.5 (8.24) | 139.7 (1.71) | 140.1 (4.24) | 139.2 (7.60) | 136.5 (1.59) | 140.9 (4.67) | 149.6 (8.73) |
| Week 72 | 122.7 (1.39) | 130.7 (3.63) | 129.2 (5.99) | 122.6 (1.46) | 128.2 (3.36) | 123.5 (5.59) | 118.0 (1.45) | 120.8 (3.76) | 113.3 (5.88) |
| Week 176 | 118.7 (2.39) | 124.5 (4.69) | 124.7 (7.58) | 117.4 (2.36) | 123.2 (3.62) | 121.9 (6.06) | 116.5 (2.14) | 122.7 (4.29) | 116.0 (7.57) |
| | | | | | | | | | |
| HDL cholesterol, mg/dL | | | | | | | | | |
| Week 0 | 47.8 (0.57) | 45.8 (1.49) | 47.7 (2.58) | 47.9 (0.57) | 46.2 (1.37) | 46.2 (2.47) | 47.5 (0.55) | 48.6 (1.58) | 44.1 (2.53) |
| Week 72 | 50.7 (0.44) | 52.2 (1.13) | 50.3 (1.81) | 51.5 (0.45) | 51.8 (1.00) | 50.9 (1.70) | 51.3 (0.46) | 51.3 (1.17) | 49.1 (1.86) |
| Week 176 | 53.8 (0.86) | 52.2 (1.53) | 52.5 (2.49) | 54.8 (1.00) | 55.2 (1.50) | 54.5 (2.50) | 55.7 (0.88) | 52.5 (1.50) | 47.4 (2.58) |
| | | | | | | | | | |
| VLDL cholesterol*, mg/dL | | | | | | | | | |
| Week 0 | 58.3 (1.19) | 58.0 (3.22) | 60.4 (5.55) | 57.3 (1.23) | 54.1 (2.86) | 62.1 (5.93) | 57.4 (1.11) | 63.2 (3.45) | 63.2 (6.24) |

| | | | | | | | | | |
|---|---|---|---|---|---|---|---|---|---|
| Week 72 | 43.0 (0.84) | 47.7 (2.28) | 46.8 (3.75) | 42.8 (0.84) | 42.6 (1.86) | 43.5 (3.28) | 39.2 (0.74) | 43.4 (2.09) | 38.3 (3.17) |
| Week 176 | 42.4 (1.41) | 45.9 (2.82) | 40.3 (4.01) | 38.3 (1.36) | 43.8 (2.27) | 41.4 (3.61) | 36.4 (1.13) | 40.5 (2.29) | 41.8 (4.51) |

*VLDL cholesterol was calculated based on triglycerides

Data are MBE (SE). Statistical analyses were performed using MMRM on log-transformed measurements with model terms of log-transformed baseline, country, sex, and treatment-by-time interaction, using Type III sum of squares. MMRM=mixed model for repeated measures; HDL = High-Density Lipoprotein; MBE=model-based estimate; LDL = Low-Density Lipoprotein; VLDL = Very Low-Density Lipoprotein; SE=standard error.

**Supplementary table 9.** Lipids at baseline, Week 72, and Week 176 in participants receiving pooled tirzepatide 5 mg, 10 mg, 15 mg by category of weight loss.

| | **Pooled tirzepatide 5 mg, 10 mg, 15 mg** | | |
|---|---|---|---|
| MBE (SE) | **<5% weight regain** | **5% to <10% weight regain** | **≥10% weight regain** |
| Triglycerides, mg/dL | | | |
| Week 0 | 127.8 (1.62) | 129.1 (4.35) | 138.4 (8.13) |
| Week 72 | 91.8 (1.05) | 97.9 (2.67) | 96.7 (4.48) |
| Week 176 | 86.1 (1.71) | 94.4 (3.11) | 92.1 (5.24) |
| | | | |
| Cholesterol, mg/dL | | | |
| Week 0 | 187.9 (1.01) | 188.0 (2.69) | 193.3 (4.83) |
| Week 72 | 174.8 (0.86) | 181.9 (2.13) | 174.9 (3.47) |
| Week 176 | 174.8 (1.44) | 179.7 (2.51) | 175.6 (4.22) |
| | | | |
| Non-HDL cholesterol, mg/dL | | | |
| Week 0 | 136.2 (1.65) | 140.7 (3.02) | 145.2 (4.82) |
| Week 72 | 123.2 (1.33) | 125.3 (2.29) | 122.0 (3.43) |
| Week 176 | 119.1 (1.47) | 122.5 (2.44) | 121.5 (4.01) |
| | | | |
| HDL cholesterol, mg/dL | | | |
| Week 0 | 46.9 (0.53) | 46.6 (0.93) | 45.8 (1.41) |
| Week 72 | 50.2 (0.39) | 51.1 (0.68) | 50.4 (1.03) |
| Week 176 | 53.9 (0.56) | 52.9 (0.87) | 52.0 (1.43) |
| | | | |
| VLDL cholesterol*, mg/dL | | | |
| Week 0 | 61.4 (1.22) | 61.1 (2.14) | 60.6 (3.28) |
| Week 72 | 44.4 (0.77) | 44.8 (1.32) | 43.4 (1.97) |
| Week 176 | 40.8 (0.84) | 44.3 (1.46) | 42.4 (2.33) |

*VLDL cholesterol was calculated based on triglycerides

Data are MBE (SE). Statistical analyses were performed using MMRM with model terms of baseline, country, sex, and treatment-by-time interaction. MMRM=mixed model for repeated measures; HDL = High-Density Lipoprotein; MBE=model-based estimate; LDL = Low-Density Lipoprotein; VLDL = Very Low-Density Lipoprotein; SE=standard error.

**Supplementary table 10.** Weight regain groups in SURMOUNT-1 compared to SURMOUNT-4.

| SURMOUNT-1 percent weight regain groups (On-treatment) | | SURMOUNT-4 percent weight regain groups (Post-Withdrawal) | | Weight regain in kg |
|---|---|---|---|---|
| Weight regain Group | Proportion of Participants | Weight regain Group | Proportion of Participants | |
| <5% | 70% | | | ~1 |
| | | <25% | 17.5% | ~2 |
| 5% - <10% | 22% | ≥25% - >50% | 25% | ~7-9 |
| ≥10% | 8% | ≥50% - >75% | 33% | ~14 |
| - | - | ≥75% | 24% | ~19 |
